# An Unusual Case of Hematochezia

**DOI:** 10.7759/cureus.8957

**Published:** 2020-07-02

**Authors:** Anna Payne, Nwe Ni Than, Rajiv Jalan, Dominic Yu

**Affiliations:** 1 Plastic and Reconstructive Surgery, Royal London Hospital, Barts Health NHS Trust, London, GBR; 2 Hepatology, Royal Free Hospital, London, GBR; 3 Radiology, Royal Free Hospital, London, GBR

**Keywords:** cecal varix, ectopic varices, variceal bleed, embolisation, tippss, hematochezia

## Abstract

Lower gastrointestinal bleeding (LGIB) is a serious and potentially life-threatening condition warranting hospital admission. The most frequent causes include diverticular disease, colitis, hemorrhoids, neoplasm, inflammatory bowel disease, and varices. Varices usually occur secondary to liver cirrhosis and are frequently located in the gastroesophageal region. Those occurring elsewhere are known as ectopic varices. The diagnosis and management of ectopic varices is challenging, and guidelines are not currently available. We report the case of recurrent large-volume hematochezia secondary to a cecal varix in a 60-year-old female with alcoholic liver cirrhosis. Initial investigation with CT angiography and endoscopy failed to identify the source of bleeding. A second CT angiogram identified a large varix in the cecum, and the patient was successfully managed with radiological embolization and transjugular intra-hepatic porto-systemic shunt (TIPSS).

## Introduction

Lower gastrointestinal bleeding (LGIB) has an annual incidence of 20 per 100,000 and is the cause of 1%-2% of hospital emergencies [1]. Portal hypertension leads to the formation varices, of which 95% are located in the gastroesophageal region [2-3]. Varices are one of the less common causes of LGIB, but are associated with significant morbidity and mortality [2]. 

Ectopic varices are defined as those occurring outside the gastroesophageal region [3]. They are responsible for 5% of variceal bleeds and carry a significant mortality rate of 40% [3]. Varices in the cecum are uncommon, with less than five cases reported in the literature [4-6]. The diagnosis and management of cecal varices is challenging, and surgical management has resulted in mortality in both reported cases [4, 6]. Endovascular intervention can be lifesaving if medical and endoscopic management are unsuccessful.

## Case presentation

A 60-year-old female with known alcoholic liver cirrhosis presented to her local hospital with sudden onset, profuse hematochezia. The blood passed per rectum was described as fresh blood with no clots. She had a background of alcoholic liver cirrhosis and an esophageal variceal bleed in 2006, which was managed with endoscopic banding. Her last alcoholic drink had been one month earlier, prior to which she drank 700 mL spirits per day. Examination revealed a soft, nontender abdomen and altered blood in the rectum.

The patient was hemodynamically unstable on presentation with a hemoglobin level of 69 g/dL, a platelet count of 52 x 109 per liter, and an international normalized ratio (INR) of 1.3. She responded to fluid resuscitation and blood products (four units of packed red cells and one unit fresh frozen plasma). CT angiography failed to identify a source of bleeding. After two days without further bleeding the patient was discharged, with a plan to perform esophagogastroduodenoscopy (EGD) and flexible sigmoidoscopy on an urgent outpatient basis.

Two weeks later the patient re-presented with a second episode of large volume hematochezia and hemodynamic instability. EGD and flexible sigmoidoscopy failed to identify the source of bleeding. Bleeding continued for three days despite treatment with tranexamic acid and blood products, after which the patient was transferred to a tertiary hepatology center for further management.

**Figure 1:**
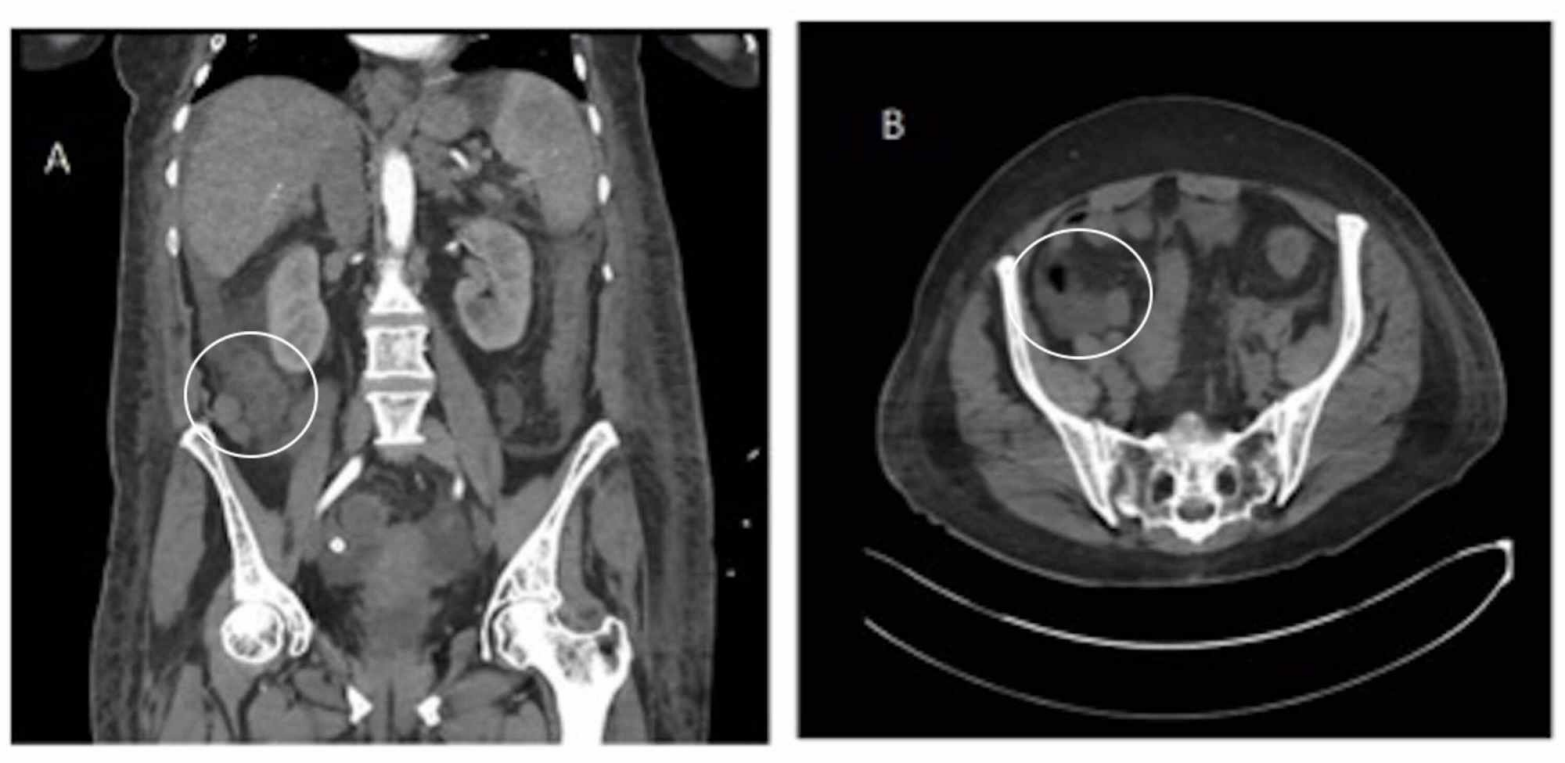
CT angiography in the venous phase. Second CT angiography shows no extravasation of contrast, indicating no active bleeding. Filling of the cecal varix (circled) is visualized in the venous phase of angiography. A: AP view. B: transverse view AP, anteroposterior

**Figure 2:**
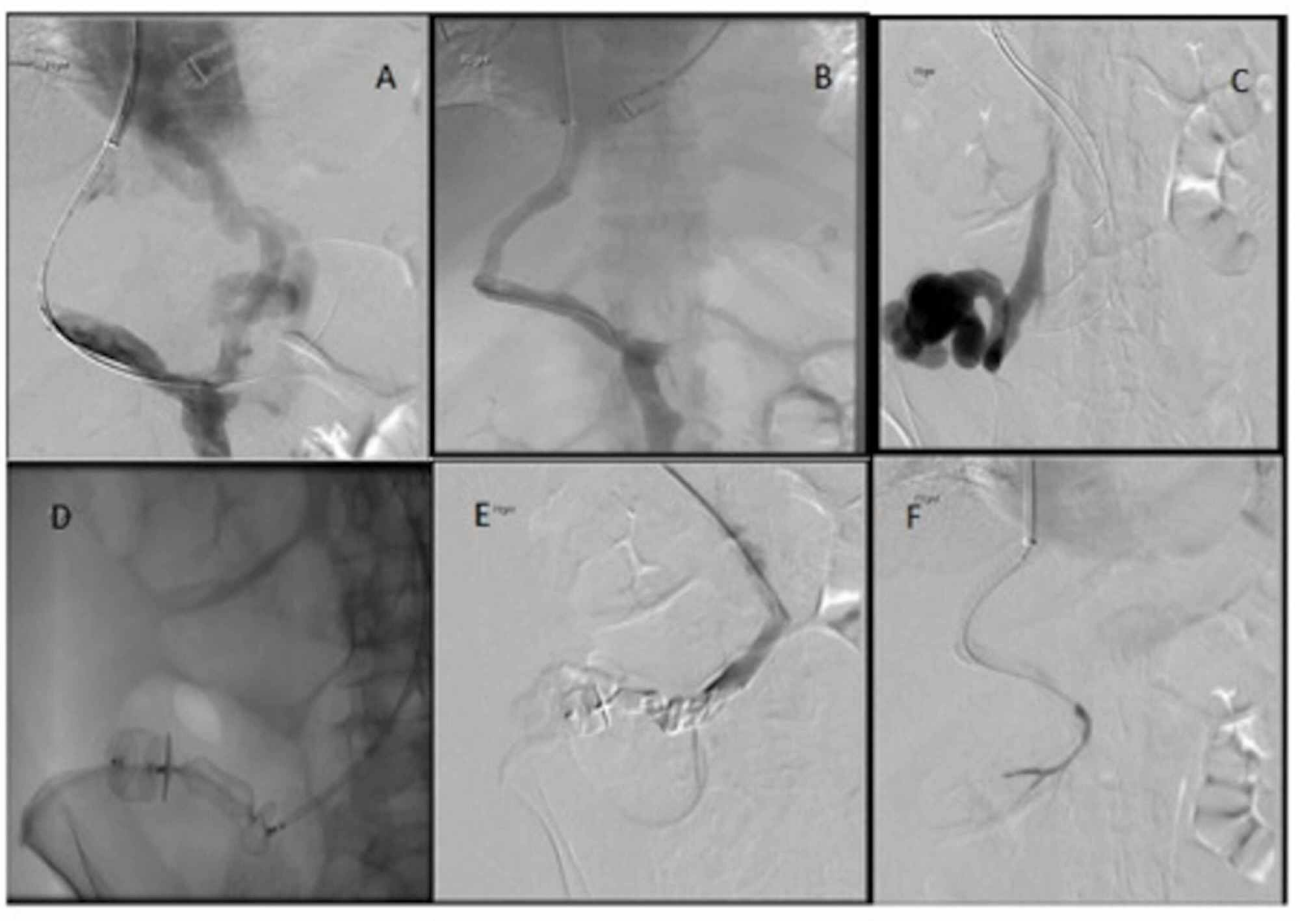
Invasive angiography, deployment of TIPSS, and embolization of the varix. A: Reversal of flow in portal vein. B: Wire passed down the superior mesenteric vein (SMV) with TIPSS deployed. C: Large cecal varix filled with contrast. D: Vascular plug deployed. E: Glue injected. F: Post-procedure demonstrating no filling of cecal varix. TIPSS, transjugular intra-hepatic porto-systemic shunt

Repeat CT angiography revealed a large varix in the cecum (Figure 1A-C). Filling of varix was visualized in the venous phase of angiography (Figures 1A-B, 2A-C) and a large collateral branch of the superior mesenteric vein was identified as supplying the varix before draining into the inferior vena cava.

The patient then underwent radiological intervention. A transjugular intra-hepatic porto-systemic shunt (TIPSS) was placed after portal pressures were found to be elevated. A 7-Fr sheath was placed in the collateral vessel just proximal to the cecal varix (Figure 2C) and two 22 mm vascular plugs were deployed followed by a small amount of glue (Figure 2D-E). No filling of the varix was seen at the end of the procedure (Figure 2F).

The patient recovered well post-procedure and suffered no further bleeding. She was discharged from hospital five days later.

## Discussion

Lower gastrointestinal bleeding is defined as bleeding occurring distal to the ligament of Treitz [1]. Varices are defined as abnormally dilated and tortuous vessels, and are most frequently caused by portal hypertension secondary to liver cirrhosis [2]. Ectopic varices make up 5% of varices and have a mortality rate of up to 40% [3]. Predisposing factors include portal hypertension, previous abdominal surgery, and congenital vascular abnormalities [5].

Cecal varices are an uncommon but potentially catastrophic cause of bleeding. Cecal varices are relatively uncommon, hence specific management guidelines are not available. The general consensus in the literature is that patients should be resuscitated followed by urgent EGD and colonoscopy. If this fails to identify a source of bleeding, angiography is indicated. Definitive treatment options include radiological or surgical intervention. Embolization of the causative vessel followed by TIPSS formation is the least invasive method and has lower one-year re-bleeding rates than embolization alone [7]. Laparotomy with bowel resection has been reported to be carried out, particularly in the context of previous abdominal surgery, but resulted in mortality in both reported cases [4, 6]. Radiological intervention is less invasive and is likely to lead to lower complication rates in patients with decompensated liver disease.

## Conclusions

Ectopic variceal hemorrhage should be considered in a patient with portal hypertension presenting with large-volume hematochezia. Following resuscitation, endoscopy should be performed to localize source of bleeding and in the absence of an obvious source, CT angiography should be performed. Radiological intervention is an effective treatment option for ectopic varices and is associated with lower morbidity and mortality than major surgical intervention. In patients with ectopic variceal bleeding, TIPSS formation in addition to vessel embolization results in lower re-bleed rates than embolization alone.
